# Supplementary material for: Prevalence of SARS-CoV-2 Omicron Sublineages and Spike Protein Mutations Conferring Resistance against Monoclonal Antibodies in a Swedish Cohort during 2022–2023
Source: Microorganisms. 2023 Sep 27;11(10):2417. doi: 10.3390/microorganisms11102417 (PMC10609123; doi:10.3390/microorganisms11102417)
Supplement: Supplementary file 1 [file microorganisms-11-02417-s001.zip › Figure S1.pdf]

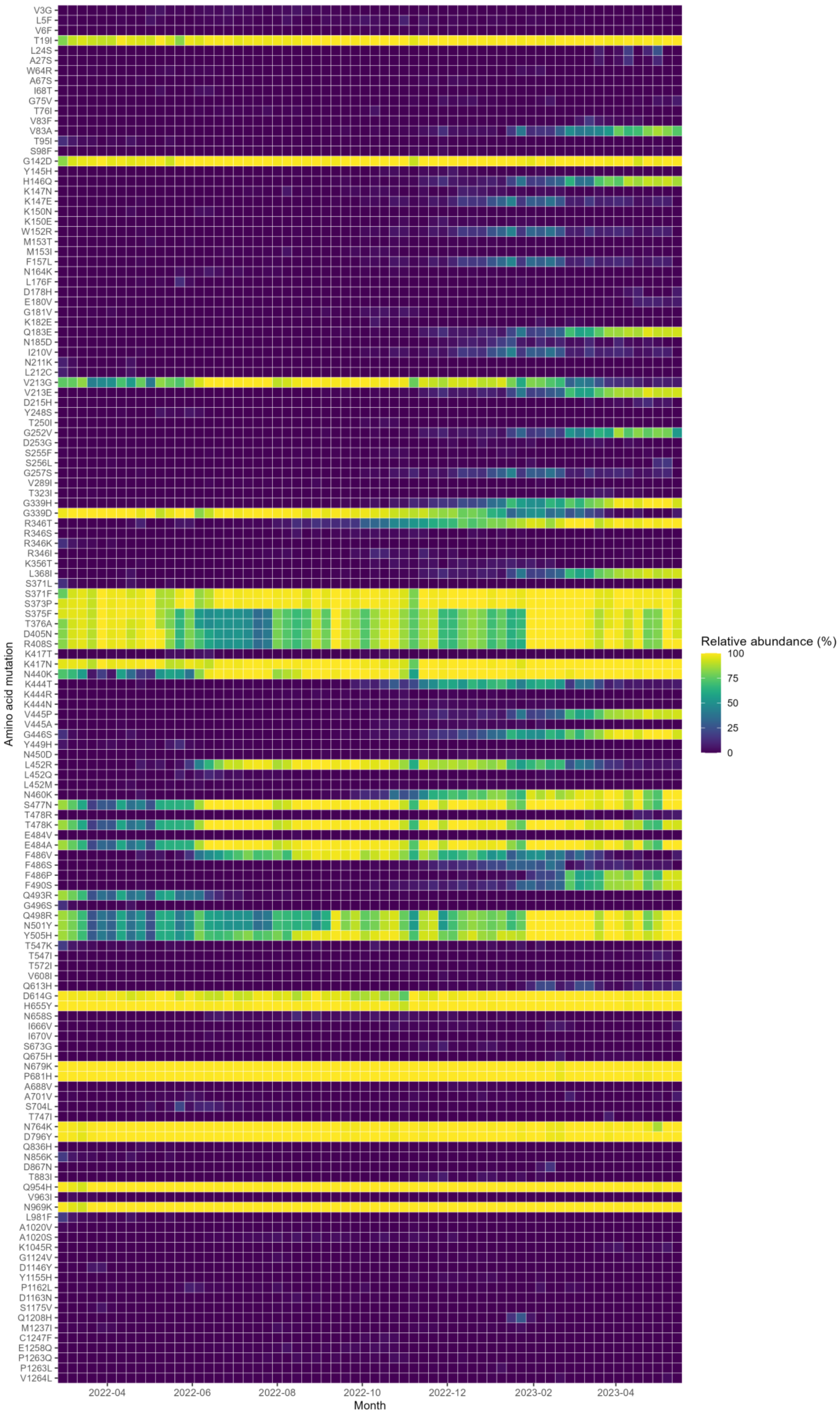

V3G
L5F
V6F
T19I
L24S
A27S
W64R
A67S
I68T
G75V
T76I
V83F
V83A
T95I
S98F
G142D
Y145H
H146Q
K147N
K147E
K150N
K150E
W152R
M153T
M153I
F157L
N164K
L176F
D178H
E180V
G181V
K182E
Q183E
N185D
I210V
N211K
L212C
V213G
V213E
D215H
Y248S
T250I
G252V
D253G
S255F
S256L
G257S
V289I
T323I
G339H
G339D
R346T
R346S
R346K
R346I
K356T
L368I
S371L
S371F
S373P
S375F
T376A
D405N
R408S
K417T
K417N
N440K
K444T
K444R
K444N
V445P
V445A
G446S
Y449H
N450D
L452R
L452Q
L452M
N460K
S477N
T478R
T478K
E484V
E484A
F486V
F486S
F486P
F490S
Q493R
G496S
Q498R
N501Y
Y505H
T547K
T547I
T572I
V608I
Q613H
D614G
H655Y
N658S
I666V
I670V
S673G
Q675H
N679K
P681H
A688V
A701V
S704L
T747I
N764K
D796Y
Q836H
N856K
D867N
T883I
Q954H
V963I
N969K
L981F
A1020V
A1020S
K1045R
G1124V
D1146Y
Y1155H
P1162L
D1163N
S1175V
Q1208H
M1237I
C1247F
E1258Q
P1263Q
P1263L
V1264L
Amino acid mutation
2022-04
2022-06
2022-08
2022-10
2022-12
2023-02
2023-04
Month
Relative abundance (%)
100
75
50
25
0

Figure S1: Heatmap showing the relative abundance of all amino acid mutations over time in the S protein of the SARS-CoV-2 from sequences collected between March 2022 and May 2023 from individuals residing in Uppsala and Örebro region.
