# Supplementary material for: Prevalence of SARS-CoV-2 Omicron Sublineages and Spike Protein Mutations Conferring Resistance against Monoclonal Antibodies in a Swedish Cohort during 2022–2023
Source: Microorganisms. 2023 Sep 27;11(10):2417. doi: 10.3390/microorganisms11102417 (PMC10609123; doi:10.3390/microorganisms11102417)
Supplement: Supplementary file 1 [file microorganisms-11-02417-s001.zip › Table S1.pdf]

Table S1. Number of sequences of each Pango lineage in May 2023.

Pango lineage	Abundance
BQ.1.25	1
CH.1.1.1	1
CH.1.1.11	2
EG.1	3
EG.1.2	5
EU.1.1	1
FK.1.2	1
FL.1	2
FL.10	1
FL.2	1
FL.2.1	1
FL.4	1
FL.5	7
FL.9	1
FU.1	1
XBB.1	2
XBB.1.16	1
XBB.1.16.1	5
XBB.1.17.1	4
XBB.1.22.2	1
XBB.1.5	13
XBB.1.5.24	12
XBB.1.5.25	1
XBB.1.5.31	1
XBB.1.5.33	1
XBB.1.5.37	3
XBB.1.5.38	1
XBB.1.5.43	1
XBB.1.5.65	1
XBB.1.9.1	18
XBB.1.9.2	2
XBB.2.3	1
XBF	3
